# Dispersion management of anisotropic metamirror for super-octave bandwidth polarization conversion

**DOI:** 10.1038/srep08434

**Published:** 2015-02-13

**Authors:** Yinghui Guo, Yanqin Wang, Mingbo Pu, Zeyu Zhao, Xiaoyu Wu, Xiaoliang Ma, Changtao Wang, Lianshan Yan, Xiangang Luo

**Affiliations:** 1 State Key Laboratory of Optical Technologies on Nano-Fabrication and Micro-Engineering, Institute of Optics and Electronics, Chinese Academy of ScienceP.O. Box 350, Chengdu 610209, China; 2 Center for Information Photonics & Communications, School of Information Science & Technology, Southwest Jiaotong University, Chengdu, Sichuan, 610031, China

## Abstract

Dispersion engineering of metamaterials is critical yet not fully released in applications where broadband and multispectral responses are desirable. Here we propose a strategy to circumvent the bandwidth limitation of metamaterials by implementing two-dimensional dispersion engineering in the meta-atoms. Lorentzian resonances are exploited as building blocks in both dimensions of the dedicatedly designed meta-atoms to construct the expected dispersion. We validated this strategy by designing and fabricating an anisotropic metamirror, which can accomplish achromatic polarization transformation in 4-octave bandwidth (two times of previous broadband converters). This work not only paves the way for broadband metamaterials design but also inspire potential applications of dispersion management in nano-photonics.

Since it was firstly illustrated by the pronounced prism experiment of Isaac Newton, the roles of chromatic dispersion in the interactions between electromagnetic wave and matter are extensively explored. It has been widely accepted that dispersion management is crucial in constructing spectrometers^1^, superprisms^2^, achromatic lens systems^3^, analog and digital optical communication system^4^. Proper dispersion contributes not only to the dense wavelength division multiplexing (DWDM) system^5^, but also the generation of soliton waves^6^.

Dispersion of natural materials is determined by the electronic and molecular energy levels, with limited tunability. In the last decades, metamaterial has emerged as revolutionary material offering unprecedented superiority for dispersion engineering, while its electromagnetic property is exclusively decided by the specific geometry and arrangement of artificial meta-atoms^7^,^8^,^9^,^10^,^11^. Nevertheless, the difficulties in manufacture impede the further development of bulk metamaterials. As two-dimensional metamaterials, metasurfaces relax the fabrication requirement and meanwhile provide plenty of exotic properties such as phase discontinuity and abnormal deflection^12^,^13^,^14^. Especially, the ability to manipulate the polarization of electromagnetic waves is sought-after for numerous applications^15^.

The past decade has witnessed the flourish of metasurface as polarization transformers owing to the miniaturized dimension and higher efficiency compared to traditional wave plates^16^,^17^,^18^,^19^,^20^,^21^. Theoretical investigations elucidate that the maximal conversion efficiency through a single metasurface can increase to 100% after introducing a reflective plane^22^. However, the highly resonant nature of meta-atoms that force the electromagnetic waves undergo a phase change ultimately causes a small bandwidth around their design frequency, as a result of the general Kramers-Kronig relations^23^.

A nascent strategy to circumvent the bandwidth limitation of polarization converters is dispersion management^15^,^24^. This concept was also widely exploited in perfect absorbers^25^,^26^,^27^,^28^,^29^ and band-pass filters^30^. Strikingly, the absorption bandwidth can be increased dramatically by taking advantage of the Drude-like dispersion of metallic thin film^28^. Despite of the great progress, there are also several shortcomings unsolved yet. On the one hand, the dispersion property was only manipulated along a single dimension of the metasurfaces^5^,^24^. Consequently, the operation bandwidth is not broad enough, typically no more than 2-octave bandwidth. On the other hand, the frequency-band selectivity of polarization manipulation as a key issue was ignored in previous studies. As a rule of thumb, a gradual transition band lays outside the working band^31^, which wastes the scarce bandwidth resource and decrease the validity. Therefore, to date, it is still a great challenge to realize an ideal one with super-octave bandwidth and excellent frequency-band selectivity.

In this paper, we demonstrated that the operation bandwidth and frequency selectivity of metasurfaces can be improved significantly with fully released dispersion management capability in two dimensions. Without the loss of generality, dispersion management of anisotropic metamaterials can be implemented via superimposing multiple Lorentzian resonances in each dimension of dedicatedly designed meta-atoms. By engineering the dispersion at orthogonal polarizations, ideal phase retardation for achromatic polarization conversion can be obtained. As an example, an achromatic circular polarization converter with 4-octave bandwidth (two times of previous broadband converters) was numerically and experimentally demonstrated. The underlying physical mechanism of dispersion engineering was explained in transfer matrix method (TMM), effective medium theory (EMT), and equivalent circuit model. The proposed scheme presents unique features including simple configuration, ultra-broadband bandwidth, and excellent frequency-band selectivity.

## Results

### Theoretical model and structure design

**Figure 1:**
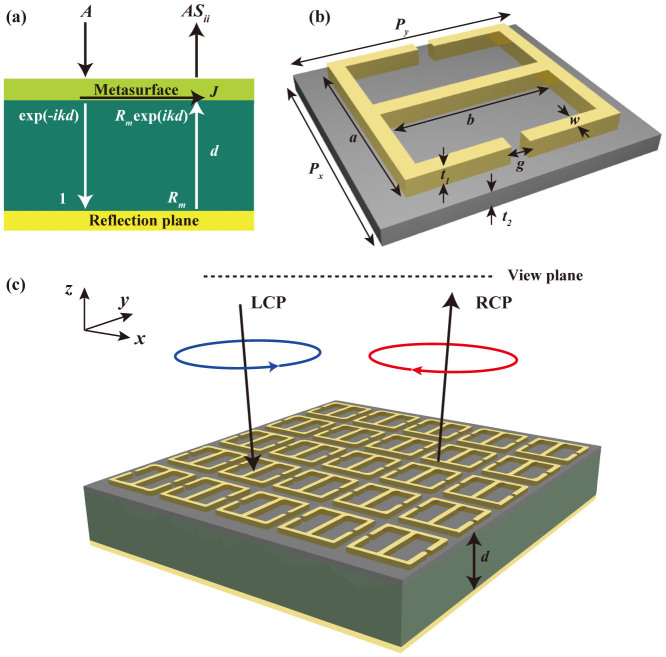
Principle of the dispersion engineering for polarization conversion. (a) Concept diagram of proposed metamirror, which is composed of a metasurface, dielectric spacer and metallic ground plane. To make the analysis as general as possible and suitable for both isotropic and anisotropic metamaterials, we take the metasurface as a thin impedance sheet without special structure, which is normally illuminated with a linear polarization. The amplitude of incident *E* field is denoted as *A* while the total reflection coefficient of the metamirror is denoted as *S_ii_*. *J* is the current flowing in the metasurface. (b) Unit cell of adopted metasurface, which is constructed by two split ring resonators sitting back to back positioned on a thin substrate. (c) Perspective view of proposed metamirror, which is able to convert the LCP waves achromatically to the RCP after reflection.

In this paper, considering no complicated anti-reflection technology is needed, we adopt a metasurface combined with a metallic reflection plane (i.e., metamirror) for polarization manipulation. To make the analysis as general as possible and suitable for both isotropic and anisotropic metamaterials, we take the metasurface as a thin impedance sheet without considering the special structure at first (Fig. 1(a)). The total reflection coefficient of the structure is analyzed by transfer matrix method (see Methods):
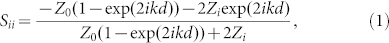
where *S_ii_*(ω) = |*S_ii_*(ω)|exp(−*jϕ_ii_*(ω)) is the total reflection coefficient for co-polarization, including the first reflection occurred at the air-metasurface interface, *i* = *x,y* represent the electric field polarized along the *x-* and *y*-direction, respectively, *Z_i_*(*ω*) represents the impedances of metasurface, *Z*_0_ = 377 is the impedance of free space, *d* is the thickness of dielectric layer, 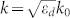 is the wave vector in the dielectric spacer. Note that TMM is equivalent to multiple interference thory^32^. Both the two methods are widely used to analyze the spectral response of multilayered structure and provide valuable guidance for dispersion management. For polarization manipulation, we optimize the phase difference Δ*ϕ*(ω) = |*ϕ_xx_*(*ω*) − *ϕ_yy_*(ω)|. For simplification, we ignored the material losses in the analyses and thus the reflection process should satisfy the energy conservation condition (i.e., |*S_xx_*(*ω*)| = |*S_yy_*(*ω*)| ≡ 1).

Before the discussion of the metasurface, we would like to clarify several vital issues for the design. Initially, the dielectric thickness *d* should be carefully selected since it will cause the phase shift origin from different physical mechanisms^13^. When the dielectric thickness is in deep-sub-wavelength, strong magnetic coupling between the meta-surface and ground plane results in nonconstant phase gradient, which is just one of the main reasons of limited bandwidth in previous devices^10^. In order to avoid strong magnetic coupling, we set the thickness of dielectric spacer *d* to 7 mm (one quarter of the wavelength at 10.7 GHz). Subsequently, low permittivity dielectric layer was exploited to ensure a gradual dispersion.

The unit cell of our metasurface is composed of two split-ring resonators (SRRs)^8^ sitting back to back, as shown in Fig. 1(b). It can be observed that the structure is asymmetric with anisotropy governed by the orientation of the unit cells. We use SRRs as electric ring resonator (ERR) instead of split-wires, I-shaped antennas and H-shaped antennas due to its more powerful dispersion management ability. Figure 1(c) schematically depicts the proposed structure normally illuminated by a left-handed circular polarization (LCP) wave, which is converted to the right-handed circular polarization (RCP) after reflection.

**Figure 2:**
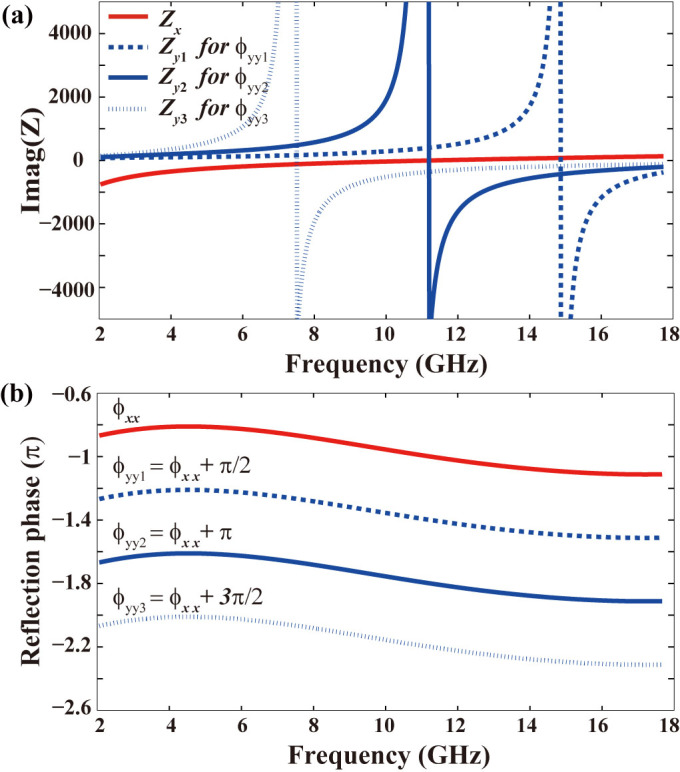
Optimal impedances for achromatic polarization conversion. (a) The given impedance *Z_x_* (red solid line) and derived optimal impedance *Z*_y_ for 1/4 wave plate (blue dash line), 1/2 wave plate (blue solid line), and 3/4 wave plate (blue dot line). The real part is zero since no material loss is considered. The region above zero is inductive and the region below zero is capacitive. (b) The given *ϕ_xx_* and derived optimal *ϕ_yy_* for achromatic 1/4, 1/2, and 3/4 wave plates.

We assumed a serial *LC* resonance with *L* = 2 nH and *C* = 100 fF for the metasurface when the electric field is along the *x*- direction. The frequency dependent impedance *Z_x_*(*ω*) = *jωL* + 1/(*jωC*) is plotted in red line in Fig. 2(a). By submitting it into equation (1), we can derive the anisotropic dispersion *ϕ_xx_*(*ω*) and *ϕ_yy_*(*ω*) with different phase differences e.g. π/2, π, and 3π/2, as shown in Fig. 2(b). Subsequently, the optimal impedance of *Z_y_*(*ω*) can be derived by submitting *S_yy_* = exp(−*jϕ_yy_*) into the equation (2):
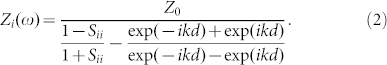
According the variation trend of *Z_y_*(*ω*) with frequency disclosed in Fig. 2(a), we determined that a parallel *LC* circuit should be constructed along the *y*-polarization. For the cases of more complex dispersion in the *x*-direction, multiple serial and parallel *LC* circuits would be needed. The relations between the circuit elements and geometrical parameters of ERR were used in the optimizing the final structure. For example, the inductor *L* is related to the current distribution in the metallic wires and increases with longer and thinner metallic structures. The capacitor *C* results from the electric field distribution in the gaps between metallic elements and tends to be larger for smaller distance between them. According to the relationship mentioned above, the geometry parameters of proposed ERRs were adjusted to approach the optimal impedance calculated by TMM. In order to obtain fine frequency-band selectivity, the impedance at the boundary of working range should deviate from the optimal impedance seriously.

### Simulation and experiment results

**Figure 3:**
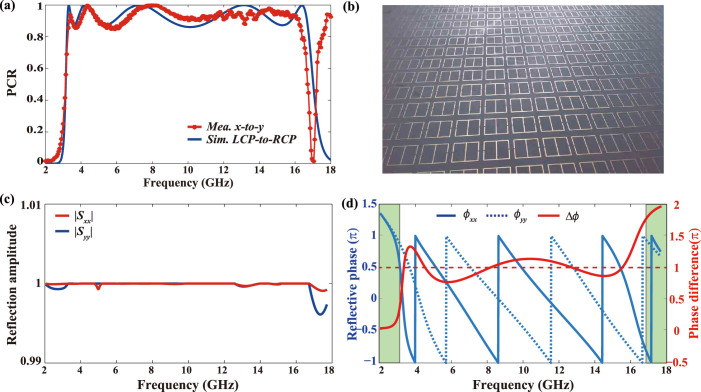
Numerical simulation and experimental verification. (a) Simulated polarization conversion ratio from LCP to RCP (blue line) and measured polarization conversion ratio from *x*- to *y*-polarization (red asterisk). (b) Photograph of the fabricated metasurface. (c) Simulated reflection amplitude of *x*- and *y*-polarization. (d) Simulated reflection phase of *x*- (blue solid line) and *y*-polarization (blue dash line), which sharing almost the same constant gradient between 3.5 GHz and 16.5 GHz. There are five intersection points between the simulated phase difference and constant π, which corresponds to the five conversion extrema in (a). In the green shaded area, the phase difference changes severely from π to 0 or 2π, corresponding to the sharp band edges.

To verify our design, a device for conversion between LCP and RCP was investigated by the finite element method (FEM) in commercial software CST Microwave Studio^TM^. In the simulations, perfect electrical conductor (PEC) model was selected for the metal patterns between 2–18 GHz, whose thickness *t*_1_ is 0.035 mm. The thin substrate under metallic patterns was chosen as *t*_2_ = 0.508 mm thick with a permittivity of 2.2 while the dielectric layer was filled with foam with permittivity equal to 1.03. Other parameters were optimized as: *P_x_* = 13.6 mm, *P_y_* = 15 mm, *a* = 11.37 mm, *b* = 10.73 mm, *w* = 0.2 mm and *g* = 0.68 mm. The simulated polarization conversion ratio (PCR) from LCP to RCP is shown in Fig. 3(a). We can see five peaks with 100% conversion ratio appear at frequencies of 3.3 GHz, 4.2 GHz, 7.3 GHz, 13.2 GHz and 16.3 GHz, respectively. The PCR is higher than 88% from 3.17 GHz to 16.9 GHz (beyond 4-octave bandwidth). Our converter is also superior to the previous devices in the frequency-band selectivity because the operation band approximates an ideal rectangle. The rectangular coefficient, defined as the bandwidth ratio between high (>80%) and low (<20%) conversion efficiency here, is high up to 0.94.

In order to prove the numerical results, a metasurface sample with outer dimension 400 × 400 mm was fabricated with print circuit board (PCB) technique as indicated in Fig. 3(b). Measurements were taken in a microwave anechoic chamber. We substituted the circular polarization conversion (LCP-to-RCP) by the linear polarization conversion (*x*-to-*y*) in the experiment since they follow the same physical mechanism (see Method). As the red asterisk in Fig. 3(a) shows, the proposed meta-mirror works well from 3.2 GHz to 16.4 GHz with efficiency higher than 85%. The near rectangular operation band behaves like an ideal pass-band filter and separates different polarizations into different frequency band. The measured spectra are in fairly good agreement with the simulated results, with discrepancies possibly arising from material loss and fabrication imperfections. High conversion efficiency occurs around 18 GHz in the measurement, which is related to high order diffraction due to the subwavelength condition is not satisfied again.

Subsequently, we investigated the anisotropic reflection coefficients under orthogonal linear polarizations. Reflection amplitudes displayed in Fig. 3(c) demonstrate |*S_xx_*| = |*S_yy_*| = 1 within the whole simulation area. Figure 3(d) reveals the reflection phases of orthogonal polarizations sharing almost the same constant phase gradient between 3.5 GHz and 16.5 GHz. As the red solid line shows in Fig. 3(d), the phase difference Δ*ϕ* fluctuates in the range of (0.73 π, 1.28 π). There are five intersection points between the practical phase difference and desired constant π, which corresponds to the five extrema mentioned above. In the green shadow area, the phase difference changes severely from π to 0 or 2π, the origination of fine frequency selectivity.

### Anisotropic impedance sheet in macroscopic model and microscopic picture

**Figure 4:**
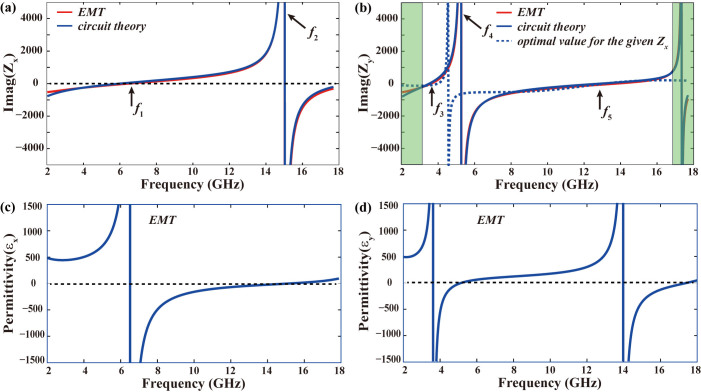
Anisotropic impedances sheet in macroscopic model and microscopic picture. (a) Effective impedance *Z*_x_ retrieved from effective material theory (macroscopic model) and electric circuit model (microscopic picture). (b) Effective impedance *Z*_y_ retrieved from effective material theory (macroscopic model) and electric circuit model (microscopic picture). Dash line in (b) shows the optimal impedance *Z*_y_ for given *Z*_x_. (c) and (d) Effective permittivity of the metasurface *ε*_x_ and *ε*_y_ retrieved from effective material theory, manifesting Loreantzian dispersion.

Macroscopic refers to the scale where the minor local field variations across the unit cells in the metamaterial regime are ignored^23^. The macroscopic model we addressed here is different from the previous effective medium theory, where the whole metamaterials are viewed as homogenous material. Here, we identify the thin metasurface as an effective impedance sheet. The anisotropic impedances were retrieved from equation (2) with the simulation results in Figs. 3(c) and (d). As Figs. 4(a) and 4(b) shows, there are three zero (*f*_1_ = 6.5 GHz, *f*_3_ = 3.6 GHz, and *f*_5_ = 13.8 GHz) and two polar points (*f*_2_ = 15 GHz, *f*_4_ = 5.3 GHz) in the operation band (3.2–16.4 GHz), implying that three serial and two parallel circuits are simultaneously formed in the ERR. The theoretical optimal impedance was also calculated and shown in Fig. 4(b), which intersects the retrieved impedance *Z*_y_ at five conversion peak points mentioned above. It is believed that better dispersion management can extend the operation band further. Note that the impedances *Z*_y_ in the bright green area were engineered to depart from the optimal impedance to obtain fine frequency selectivity. Such film can also be interpreted from the effective permittivity *ε_eff_*, which is directly related to the sheet impedance^25^:
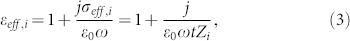
where *σ_eff,i_* = 1/(*tZ_i_*) is the effective complex conductivity and *t* is the thickness of the metamaterial film. The anisotropic permittivity *ε_eff_*_, *x*_ and *ε_eff_*_, *y*_ was calculated and displayed in Fig. 4(c) and Fig. 4(d), which demonstrate that the expected dispersion are constructed by superimposing multiple Lorentzian functions^33^.

**Figure 5:**
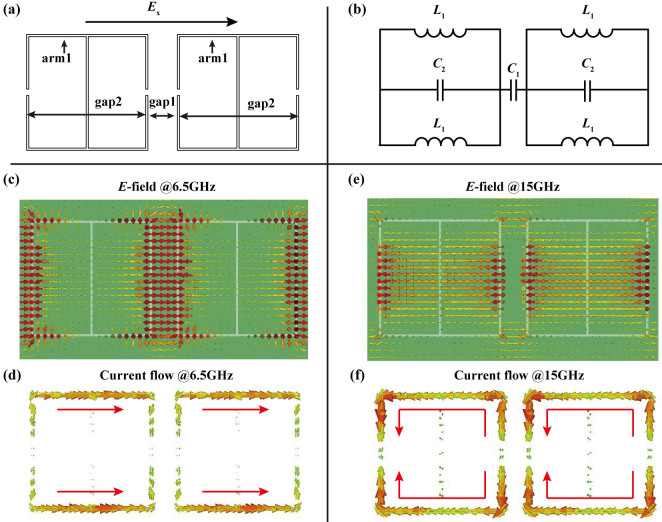
Circuit model of the proposed structure under the *x*- polarization. (a) Origination of the inductor and capacitor when the electric field is polarized along the *x*-direction. (b) Equivalent circuit model for the *x*-polarization. (c) Electric field distributions and (d) volumetric current flows at *f*_1_ = 6.5 GHz (zero point of Z_x_). (e) Electric field distributions and (f) volumetric current flows at *f*_2_ = 15 GHz (polar point of Z_x_).

Subsequently, the impedance sheet was examined in microscopic picture. We consider two horizontally neighboring unit cells when the incident electric field is along the *x*- direction, as shown in Fig. 5(a). In this case, arm1, gap1, and gap2 behave as inductors and capacitors, with equivalent circuit as shown in Fig. 5(b). According to the classic circuit theory, the frequency-dependent impedance can be expressed as:
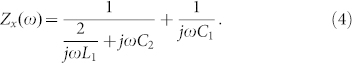
Then we examine the electric field distribution and volumetric current flow at frequencies *f*_1_ and *f*_2_. The simulation results in Figs. 5(c–f) showed that the electric field is confined in gap1 at *f*_1_ and gap2 at *f*_2_. At *f*_1_ the circuit is dominated by a serial circuit while a parallel circuit plays a more profound role at *f*_2_.

**Figure 6:**
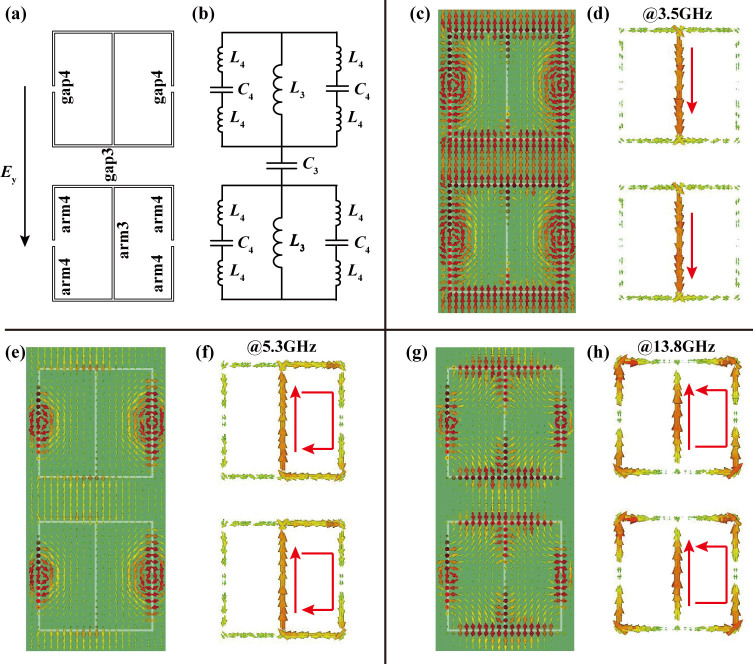
Circuit model of the proposed structure under the *y*-polarization. (a) Origination of the inductor and capacitor when the electric field is polarized along the *y*-direction. (b) Equivalent LC circuit model for the *y*- polarization. (c) Electric field distributions and (d) volumetric current flows at *f*_3_ = 3.5 GHz (zero point of Z_y_). (e) Electric field distributions and (f) volumetric current flows at *f*_4_ = 5.3 GHz (polar point of Z_y_). (g) Electric field distributions and (h) volumetric current flows at *f*_5_ = 13.8 GHz (zero point of Z_y_).

When the incident electric field is along the *y*-direction we consider two vertically neighbor unit cells, as shown in Fig. 6(a). The arm3, arm4, gap3, and gap4 behave as inductors and capacitors, with equivalent circuit as shown in Fig. 6(b). The frequency-dependent impedance is:
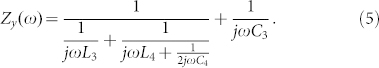
The electric field distribution and volumetric current flow at frequencies *f*_3_, *f*_4_, and *f*_5_ were depicted in Figs. 6(c–h). At these frequencies, the whole circuit is dominated by serial, parallel, and serial circuit, respectively, which cause the impedances arrive to zero or pole at these frequencies. To check the consistency between the microscopic picture and macroscopic model, the circuit parameters must be evaluated by fitting the impedances with that retrieved from *S*-parameters. When theses circuit parameters satisfy (*L*_1_, *C*_1_, *C*_2_) = (5 nH, 200 fF, 45 fF) and (*L*_3_, *C*_3_, *L*_4_, *C*_4_) = (11 nH, 100 fF, 0.1 nH, 41 fF), the microscopic picture (blue line) agrees well with the macroscopic model (red line), indicating that our physical model is robust.

## Discussions

In summary, we have demonstrated that the operation bandwidth of metasurfaces can be extended significantly with two-dimension dispersion management of the subwavelength constitutes. To verify this proposal, a circular polarization converter was numerically and experimentally demonstrated. Nearly 100% conversion ratio is realized at frequencies of 3.3 GHz, 4.2 GHz, 7.3 GHz, 13.2 GHz and 16.3 GHz, respectively. The PCR is higher than 85% in frequencies ranging from 3.2 GHz to 16.4 GHz (i.e., 4-octave bandwidth). Moreover, the operation band has a rectangular coefficient high up to 0.94, which is superior to previous metamaterial converters regarding the frequency-band selectivity.

To ensure causality, we have used Lorentz-type dispersion as building blocks to develop the target dispersion. It is worth noting that larger bandwidth is feasible by taking advantages of other types of dispersion. Owing to the scalability of Maxwell's equations, the structure can be scaled to higher frequencies such as Terahertz and near infrared. In addition, this approach may inspire the applications of dispersion management in integrated photonics.

## Methods

### Transfer matrix method analysis

As shown in Fig. 1(c), a plane wave is normally projected on the metamirror with the amplitude of *E* field denoted as *A*. Due to reflections occur at the meta-surface and background plane, both the forward and backward going waves exist in the dielectric spacer and surrounding space^27^. In order to utilize transfer matrix method, we assume the amplitude of *E* field of forward (backward) going wave at the reflection plane is 1 (*R_m_*). While the reflectivity the meta-surface is represented as *S_ii_*. According to the boundary conditions of the Maxwell's equations, electromagnetic fields at the upper side (*E^U^* and *H^U^*) and lower side (*E^L^* and *H^L^*) of meta-surface should satisfy:
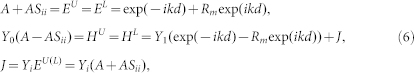
where *Y_0_*, *Y_i_*, 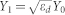 and 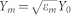 are the intrinsic the intrinsic admittance of vacuum, meta-surface, dielectric spacer and metal, *ε_d_* and *ε_m_* are permittivity of dielectric and metal, *k_0_* and 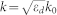 are the wave vectors in the vacuum and dielectric spacer, *R_m_* = (*Y*_1_ − *Y_m_*)/(*Y*_1_ + *Y_m_*) is the reflection coefficient at the ground plane, *J* is the surface current flowing in the metasurface. By eliminating *A* in above equations, one can obtain the impedance of meta-surface and reflection coefficient. For simplicity, assuming the dielectric layer is free space with permittivity *ε_d_* = 1 and the metal is perfect electric conductor (PEC) with *ε_m_* = ∞, we can derive *R_m_* = −1. Consequently, the impedance and reflection coefficient can be expressed as:
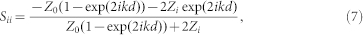
and
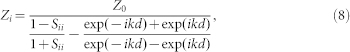
where *i* = *x,y* represent the electric field polarized along *x* and *y* direction, respectively.

### Measurements

The measurements were carried in a microwave anechoic chamber. Two standard linearly polarized horn antennas (the electric field is parallel to *x* axis) as transmitter and receiver, respectively, were connected to the two ports of a vector network analyzer R&S ZVA40. The incident angle was set as 5°, which is a good approximation of the normal incidence. The sample was rotated 45° so that the *x*-polarized wave is transformed to the *y*-polarization.

## Author Contributions

Y.H.G., Y.Q.W. and M.B.P. contributed equally to the numerical simulation and physical interpretation. Z.Y.Z., X.Y.W., X.L.M. and C.T.W. fabricated the sample and carried out the experiment, Y.H.G., M.B.P. and L.S.Y. wrote the manuscript. X.G.L. conceived the original idea and supervised the project. All the authors have analyzed and discussed the results thoroughly and contributed to the writing of the manuscript.
